# Altered glycosylation of MUC1 influences its association with CIN85: the role of this novel complex in cancer cell invasion and migration

**DOI:** 10.18632/oncotarget.1265

**Published:** 2013-09-07

**Authors:** Sandra Cascio, Adam M. Farkas, Rebecca P. Hughey, Olivera J. Finn

**Affiliations:** ^1^ Department of Immunology, University of Pittsburgh School of Medicine, Pittsburgh, PA, 15261; ^2^ Fondazione Ri.Med, via Bandiera, Palermo, 90133, Italy; ^3^ Department of Medicine, Renal-Electrolyte Division, University of Pittsburgh School of Medicine, Pittsburgh, PA 15261

**Keywords:** MUC1/CIN85 complex, protein-protein interaction, cell migration, invadopodia-like structures, cancer metastasis

## Abstract

MUC1 is a transmembrane glycoprotein abnormally expressed in human adenocarcinomas. The extracellular domain of MUC1 contains a variable number of tandem repeats (VNTR) region that is extensively O-glycosylated in normal epithelia and underglycosylated in tumor cells. This change in posttranslational modification of MUC1 leads to changes in its normal functions including, we hypothesized, its interaction with other molecules. We identified CIN85, an adaptor protein involved in multiple cellular processes including signal transduction, cytoskeletal remodeling and cancer cell invasion, as one of several proteins that associate with MUC1 in tumor cells. CIN85 associates with both the cytosolic tail and the extracellular VNTR of MUC1. Co-immunoprecipitation and confocal immunofluorescence confirmed that MUC1 and CIN85 co-localize primarily at the plasma membrane but the complex can be found also in the cytosol and on the cytoskeleton. MUC1 and CIN85 are both over-expressed in early as well as advanced clinical stages of breast cancer and co-localize on invadopodia-like structures implicated in cell invasion. siRNA-mediated silencing of CIN85 and/or MUC1 revealed that MUC1 enhances CIN85-dependent breast cancer cell migration and invasion *in vitro*. However, ectopic expression of MUC1 enhances the motility induced by CIN85. When tested *in vivo* in a tumor metastasis model of B16 melanoma, mice injected with CIN85-depleted melanoma cells exhibited few or no lung metastasis and, similarly to the *in vitro* results, overexpression of MUC1 recovered the shCIN85-reduced metastatic process. Our findings implicate this newly identified CIN85/MUC1 complex associated with invadopodia-related molecules in promoting the invasive and metastatic potential of breast cancer.

## INTRODUCTION

Mucin 1 (MUC1) is a transmembrane glycoprotein normally expressed at low levels on the apical surface of epithelial cells. In most adenocarcinomas and in other inflammatory diseases of epithelial tissues, MUC1 is overexpressed and loses the polarity of its expression. The large extracellular domain contains the variable number of tandem repeats region (VNTR) composed of around 20-200 tandem repeat (TR) units of 20 amino acids each, and is rich in prolines and O-glycosylated serines and threonines. The extracellular domain is non-covalently linked to the smaller subunit consisting of an autocatalytic cleavage SEA-module, the transmembrane domain (TM) and the cytoplasmic tail (CT). On normal epithelia, MUC1 VNTR are extensively O-glycosylated with long branched glycans, whereas on tumor cells they are markedly hypoglycosylated with simpler and shorter glycan chains. Elevated levels of MUC1 on the tumor have been associated with invasiveness and poor prognosis in colon, pancreas, breast and bladder cancer [1,2]. There is additional evidence that MUC1 contributes to the growth and metastatic properties of tumors [3-5]. The cytoplasmic tail of MUC1 (MUC1.CT) was reported to be involved in a wide range of intracellular signaling processes by associating with several protein partners and modulating their function. Recently we showed that the extracellular domain and the VNTR also participate in intracellular signaling by activating the NF-κB pathway [6]. In addition to higher levels of MUC1 in tumors compared to normal cells, hypoglycosylation of VNTR is the most important characteristic of tumor MUC1. Fewer sugars on the VNTR increase the accessibility of the peptide backbone to more efficient or completely new protein-protein interactions that can profoundly change intracellular signaling in tumors compared to normal cells [7,8]. We investigated what proteins in tumor cells associate with MUC1 and how those associations affect various tumor cell functions. Here we show an important new association between CIN85 and MUC1 in tumor cells that involves both the cytoplasmic tail and the extracellular domain of MUC1.

CIN85 was first identified in human cells as a Cbl-interacting protein of 85 kDa [9]. c-Cbl is a multi-adaptor-associated ubiquitin ligase that recruits CIN85 and initiates endocytic internalization, trafficking and sorting of various molecules, such as several activated receptor tyrosine kinases (RTK) including the epidermal growth factor receptor (EGFR) [10,11]. CIN85 contains three Src homology 3 (SH3) domains at its N-terminus followed by a proline-rich region and a C-terminal coiled-coil region [12]. SH3 domains are small modular interaction domains that generally bind to proline-containing targets (PxxP motifs). It has been shown that SH3 domains of CIN85 recognize an atypical proline–arginine motif, PXXXPR, in Cbl and many other molecules such as SHIP-1, Hip1R, p115RhoGEF, ASAP1 and ARAP3 that are implicated in the control of clathrin-mediated receptor endocytosis, receptor recycling, and cytoskeletal rearrangements [10–12]. This is of interest because each repeat in the MUC1 VNTR contains a highly conserved sequence PDTRPA, which is a good candidate for binding of CIN85. CIN85 has been also implicated in a number of important cellular processes including signal transduction, vesicle-mediated transport, cytoskeleton remodeling, immunological synapse, cell migration and invasion [11,13–16]. Recently, CIN85 was reported to be detected on lamellopodia and invadopodia which are involved in cell adhesion and migration, suggesting that overexpression of CIN85 could promote invasiveness of cancer cells [17].

We report here a newly identified association of CIN85 with the hypoglycosylated tumor form of MUC1, which affects the functions of both molecules. Subcellular protein fractionation revealed that CIN85 and MUC1 co-localize mainly at the plasma membrane but also in the cytosol and on the cytoskeleton. By associating with CIN85 on invadopodia, MUC1 regulates its role in cell migration and invasion. Abnormal expression of either molecule or both (as seen in cancer cells) allowing this association can profoundly disturb processes that each molecule alone regulates in normal cells.

## RESULTS

### Novel association of CIN85 and MUC1

**Figure 1:**
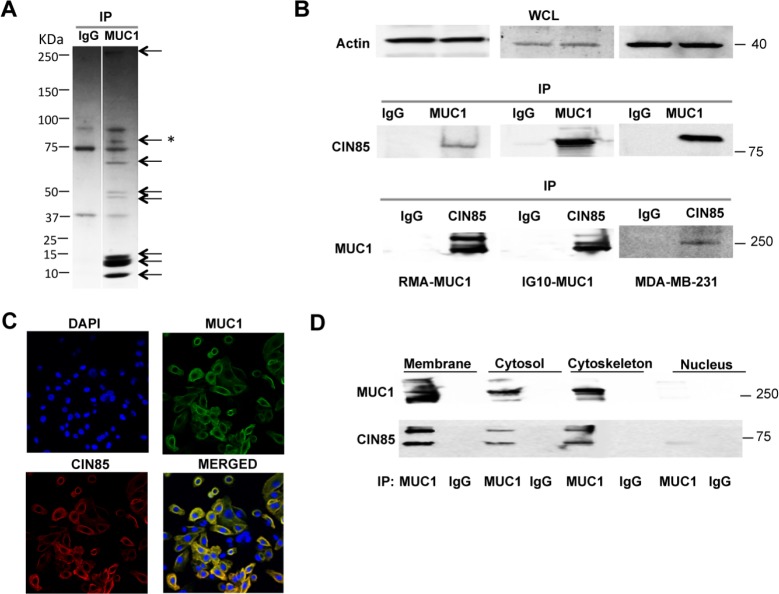
MUC1 association with CIN85 A) SDS-PAGE of the MUC1/22TR-transfected RMA cells immunoprecipiated with anti-MUC1 antibody 3C6 or control IgG. The * marks the band that yielded the CIN85 peptide; B) lysate proteins from MUC1-transfected RMA or IG10 cells, and from MDA-MB-231 cells were immunoprecipitated with anti-MUC1 antibody and immunoblotted with anti-CIN85 antibody. Reverse control was performed: CIN85-immunoprecipitated proteins were immunoblotted with MUC1 antibody; immunoblotting (WCL) of whole cell lysates for beta-actin was carried out as a control C) Confocal immunofluorescence microscopy with FITC-labeled mouse anti-human MUC1 antibody CD227 (green) anti-CIN85 antibody (red). Nuclei were stained with DAPI (blue) D) Subcellular fractions from IG10-MUC1 cells were immunoprecipitated with anti-MUC1 3C6 antibody and immunoblotted with anti-CIN85 and anti-MUC1 Ab5 antibody.

To identify proteins that interact with MUC1 responsible for carrying out important functions in tumor cells, we transfected the mouse tumor cell line RMA with cDNA coding for full-length MUC1 containing 22 tandem repeats in the VNTR domain (RMA-MUC1). We precipitated cell lysates from the transfected cells with the anti-MUC1 antibody 3C6 specific for the VNTR, or with a control mouse IgG. We visualized by SDS-PAGE and Coomassie Brilliant Blue staining all the proteins that co-precipitated with MUC1. Several bands were observed (Fig. 1A) and isolated from the gel for further identification by sequencing. The sequence of one of the bands in the 75-100 kDa range corresponded to the sequence of CIN85. In repeated coprecipitation experiments we confirmed this CIN85/MUC1 association in RMA-MUC1 by showing their coprecipitation from the MUC1-transfected mouse ovarian tumor cell line IG10-MUC1 and the human breast cancer cell line MDA-MB-231 that naturally overexpresses MUC1 (Fig. 1B). Whole cell lysates were immunoprecipitated with anti-MUC1 antibody, the precipitates run on an SDS gel and immunoblotted with anti-CIN85 antibody. There was a clear band in the MUC1 immunoprecipitates from all the cell lines in the CIN85 molecular weight range as detected by anti-CIN85 antibody. The association of these two molecules was further confirmed by blotting CIN85 immunoprecipitates with anti-MUC1 antibody against the VNTR epitopes (Fig.1B). Immunoblotting with anti-actin antibody served as control for equal sample loading.

We used confocal immunofluorescence microscopy (Fig. 1C) to determine where MUC1 and CIN85 might be interacting within the cell. Staining with anti-MUC1 and anti-CIN85 antibodies showed co-localization of MUC1 and CIN85 both intracellularly and on the cell surface. Immunoprecipitation with anti-MUC1 antibody of subcellular fractions derived from IG10-MUC1 cells and immunoblotting with anti-CIN85 and anti-MUC1 antibodies showed that MUC1 and CIN85 co-localize in the membrane (includes plasma membrane, mitochondria, Golgi, endosomes and endoplasmic reticulum), the cytosol and the cytoskeleton fractions, with very little or none detected in the nuclear fraction (Fig. 1D).

**Figure 2:**
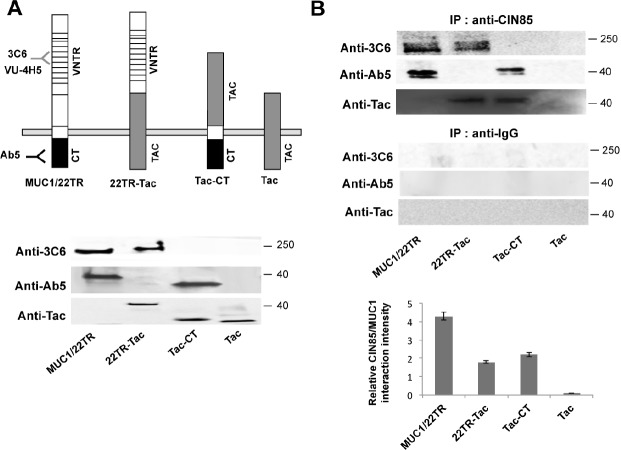
CIN85 associates with two distinct MUC1 domains A) *Upper panel*. Schematic of MUC1 forms expressed in transfected cells: MUC1/22TR: full length MUC1 containing 22 tandem repeats in the VNTR domain; 22TR-Tac: chimera containing the ectodomain of MUC1 and the transmembrane and cytoplasmic domains of IL-2 α receptor subunit (Tac); Tac-CT: chimera containing the ectodomain of Tac and the cytoplasmic tail of MUC1; Tac: full length IL-2 α receptor subunit. *Lower panel*. Whole cell lysate from MDCK cells stably transfected with MUC1/22TR, 22TR-Tac, Tac-CT and Tac was immunoblotted with anti-MUC1 antibodies Ab5 (anti-CT), 3C6 (anti-VNTR) and anti-Tac, as positive control B) Whole cell lysate from MDCK cells stably transfected with MUC1/22TR, 22TR-Tac, Tac-CT and Tac was immunoprecipitated with anti-CIN85and immunoblotted with Ab5, 3C6 or Tac antibodies. The relative MUC1/CIN85 interaction intensity was calculated by Image J.

To determine which of the two MUC1 subunits interacts with CIN85, we used Madin Darby Canine Kidney (MDCK) epithelial cells transfected to express either the whole MUC1 molecule with 22 tandem repeats (MUC1/22TR), the chimera containing the ectodomain of MUC1/22TR, the transmembrane and cytoplasmic domains of Tac (Interleukin-2 receptor α-subunit) (22TR-Tac), or the chimera prepared with the ectodomain of Tac and the MUC1 transmembrane and cytoplasmic domains (Tac-CT). Cells expressing only Tac were used as a negative control (see schematic models in Fig. 2A upper panel). Expression levels of MUC1/22TR, 22TR-Tac, Tac-CT and Tac were analyzed by Western Blotting of the whole cell lysates (Fig. 2A lower panel). These various cell lysates were immunoprecipitated with anti-CIN85 antibody, resolved on SDS-PAGE and then immunoblotted with either anti-MUC1 antibody Ab5 that recognizes the cytoplasmic tail, antibody 3C6 that recognizes the VNTR domain and anti-Tac antibody. Immunoprecipitates conducted with an unrelated IgG served as negative controls (Fig. 2B). Both 22TR-Tac (lacking the MUC1 cytoplasmic tail), as well as Tac-CT (lacking the MUC1 extracellular domain) were able to co-precipitate with CIN85. The whole MUC1/22TR molecule co-precipitated more CIN85 than MUC1 constructs lacking either the extracellular domain (2.4-fold) or the cytoplasmic tail (1.9-fold). We confirmed this finding in the human melanoma cell line DM6, mouse melanoma cells B16 and mouse lymphoma RMA, that were stably transfected with either MUC1/22TR or MUC1/Y, an isoform of MUC1 lacking most of the extracellular domain. Our results show that the presence of the extracellular domain increased significantly co-precipitation of MUC1 and CIN85 (Supplementary Fig S1).

### Altered glycosylation of MUC1 in tumors facilitates its interaction with CIN85

**Figure 3:**
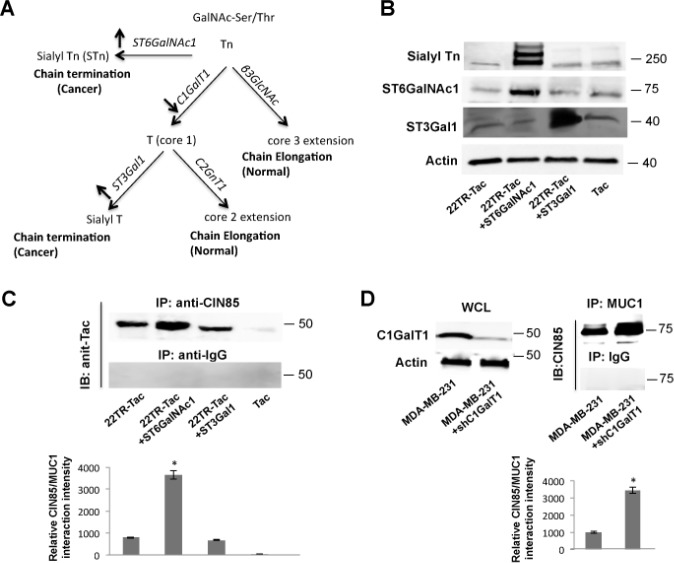
Hypoglycosylation of MUC1 facilitates the interaction between MUC1 and CIN85 A) Pathway of the Core O-glycan biosynthesis. The enzyme expressions manipulated in this study are reported in bold character next to the arrows. B) WCL from MDCK cells stably transfected with ST6GalNAc-1, ST3Gal1, and/or 22TR-TAC or only Tac was immunoblotted with anti-ST3Gal1, anti-ST6GalNAc1, anti-sialyl Tn and anti-actin C) or immunoprecipitated with anti-CIN85 (A-7) or control IgG and immunoblotted with anti-Tac D) MDA-MB-231 cells were transfected with shRNA for C1GalT1 and cell lysetes were immunoprecipitated with anti-MUC1 antibody and immnublotted with CIN85. The left panel shows C1GalT1expression by immunoblotting whereas actin was used as loading control.

As the VNTR of MUC1 is severely underglycosylated in tumors compared to normal cells, we wanted to know if these changes in O-linked glycosylation promote de novo or stronger interactions of MUC1 with CIN85. To profoundly inhibit MUC1 glycosylation in MDCK/22TR-Tac cells, we overexpressed CMP-Neu5Ac GalNAc α2,6-sialyltransferase (ST6GalNac-1) or the enzyme ST3 β-galactoside α-2,3-sialyltransferase 1 (ST3Gal1). ST6GalNac-1 prevents O-glycan elongation by adding a sialic acid residue in α2,6-linkage to GalNAc-O-Ser/Thr, a carbohydrate structure known as sialyl-Tn antigen [20,21]. ST3Gal1 adds a sialic acid in α2,3-linkage to Galβ1,3GalNAc, structure known as Sialyl-T antigen (see model in Fig. 3A). The ST6GalNAc-I and ST3Gal1 expression was confirmed by Western Blotting (Fig 3B). Normal, low levels were detected in untransfected cells whereas ST6GalNac1 or ST3Gal1 transfection resulted in 4-fold higher expression in 22TR-Tac+ST6GalNac1 or 22TR-Tac+ST3Gal1 cells. We also detected the expression of the abnormally shortened carbohydrate antigens by using anti-sialyl Tn antibody (Fig. 3B). The Sialyl-Tn-antigen was significantly increased (5-fold) in ST6GalNac-1 transfected cells. Immunoprecipitated with anti-CIN85 antibody and immunoblotting with anti-Tac antibody showed a larger amount of co-precipitation of MUC1 and CIN85 in O-linked glycosylation inhibited 22TR-Tac+ST6GalNac1 cells compared to control cells (Fig. 3C). No significant difference was found in ST3Gal1-transfected cells compared to 22TR-Tac-transfected cells (Fig. 3C). This suggested that Tn-MUC1 containing a single GalNac on the serines and threonines could better promote the interaction with CIN85 compared to T-MUC1 with two sugars (GalNac-Gal). A further examination of the effect of O-glycosylation on MUC1 and CIN85 interactions, was done by knocking-down the expression of C1GalT1, a core 1 β 1,3-galactosyltrasnferase, responsible for conversion of Tn antigen to T antigen (see model in Fig 3A). The resulting core 1 precursor for all can be elongated to the branched core 2 by the action of core 2 β 1,6-acetylglucosaminyltransferase (C2GnT) or sialyated by ST3Gal1 forming sialyl T antigen (Fig 3A). Transfection of C1GalT1 shRNA into MDA-MB-231 MUC1+ breast tumor cells decreased C1GalT1 protein level by 90% compared with untransfected cells (Fig 3D). When anti-MUC1 immunoprecipitates from MDA-MB-231+shC1GalT1 lysates with were immunoblotted with anti-CIN85, we observed that down-regulation of C1GalT1 enhanced the amount of co-precipitated CIN85 (Fig 3D). Taken together, our data revealed that the two forms of tumor-associated, hypoglysylated Tn- and sialyl Tn-MUC1 are the optimal partner for CIN85.

### CIN85 and MUC1 co-localize in primary human breast tumors on invadopodia-like structures where they affect invasion and migration

**Figure 4:**
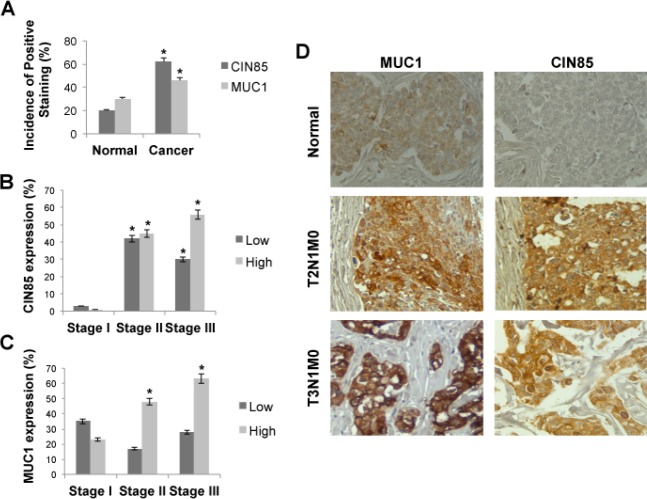
CIN85 and MUC1 are overexpressed in advanced stages of breast cancer A) Graph represents the percentage of CIN85 and MUC1 positive staining on normal and breast cancer tissue. B,C) Percentage of breast tumors in each stage that contained low or high levels of CIN85 or MUC1. D) Immunohistochemistry staining of MUC1 and CIN85 in normal tissue (upper), and breast cancer tissue at stage T2 (middle) and stage T3 (bottom). Original magnification is x200. The tumors were classified according to the TNM systems of Elston and Ellis (T: invasion of primary tumours, N: metastasis to regional lymph nodes, M: metastasis to distal organs) as well as the guide lines from American Cancer Society.

Since the MUC1-CIN85 interactions described above were pursued in detail and confirmed in mouse and human cell lines that could be experimentally manipulated and controlled, we wanted to determine if these interactions were occurring *in vivo* in human tumors. We chose to examine a human breast cancer tissue microarrays (TMA) that contained normal (5 cases) and malignant (invasive ductal) (33 cases) tissues, with each sample in duplicate. Both CIN85 and the tumor form of MUC1 were expressed at significantly higher levels in invasive ductal carcinoma sections compared with normal epithelium (Fig. 4A). CIN85 was expressed at low level in early stage tumors (stage I), but at a significantly higher levels in stage II and III (Fig. 4B and D). Similarly, MUC1 was markedly overexpressed in cancer compared to normal tissue, especially in advanced stages of disease (Fig. 4C and D).

**Figure 5:**
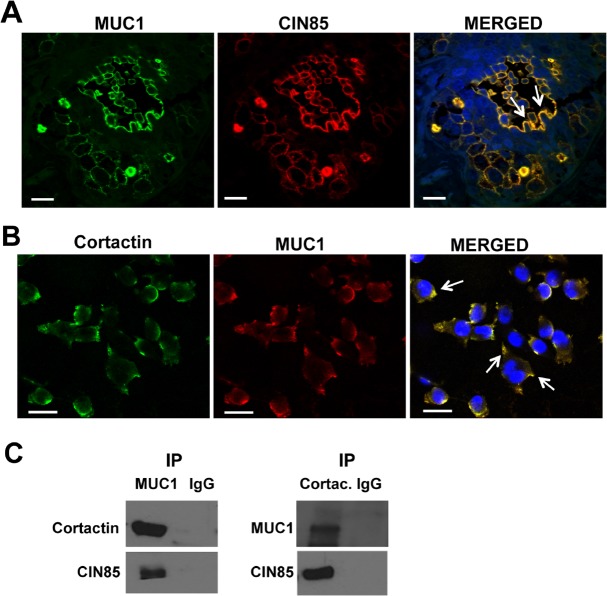
CIN85 and MUC1 colocalize in invadopodia-like protrusion structures in breast cancer Confocal immunofluorescence microscopy: Tissue samples were fixed and stained with FITC-labeled mouse anti-human MUC1 antibody CD227 (green), anti-CIN85 antibody (red) in (A). MDA-MB-231 breast cancer cells were fixed and stained with anti-MUC1 (4H5) (red), and anti-cortactin (green) (B). Nuclei were stained with DAPI (blue). Cell lystes from MDA-MB-231 were immunoprecipitated with anti-MUC1 (left panel), anti-cortactin (right panel) or control anti-IgG. MUC1-immunoprecipitated proteins were immunoblotted with anti-cortactin and anti-CIN85 antibodies, Cortactin-immunoprecipitated proteins were immunoblotted with anti-MUC1 and anti-MUC1 (C). Bar, 25 μm. Magnification 60X

Next, we investigated by confocal immunofluorescence microscopy whether MUC1 and CIN85 co-localized in these tissue samples. CIN85 and MUC1 colocalized in 21 specimens of 38 sections analyzed and in many they were also found on large invadopodia-like protrusions (Fig. 5A, white arrows). CIN85 has been previously reported to participate in the formation of lamellopodia and invadopodia structures [17]. In order to confirm the identity of these structures, we looked for the presence of cortactin, a regulator of podosome/invadopodia formation by its association with actin-regulatory proteins. MDA-MB-231 cells were treated with EGF which stimulates the formation of invadopodia in carcinoma cells [22]. Confocal microscopy immunofluorescence and co-immunoprecipitation assay show that all three molecules, CIN85, MUC1 and cortactin, co-localize in MDA-MB-231 cells (Fig. 5B). In Fig. 5C, cell lysates from MDA-MB-231 were immunoprecipitated with anti-MUC1 antibody (left panel) or anti-Cortactin antibody (right panel) and immunoprecipitated proteins immunoblotted with anti-Cortactin and anti-CIN85 antibodies or anti-MUC1 and anti-CIN85 antibodies. Each individual precipitate brought down all three molecules.

We also investigated colocalization of MUC1 and CIN85 with another component of invadopodia, membrane type 1 metalloprotease (MT1-MMP). The key difference between invadopodia and other types of protrusions, such as filopodia and lamellipodia, is that invadopodia degrade the extracellular matrix and they require delivery of vesicles containing MT1-MMP. As showed in Supplemental Figure 2, MUC1 was found together with MT1-MMP at the leading edge of the MDA-MB-231 tumor cells.

**Figure 6:**
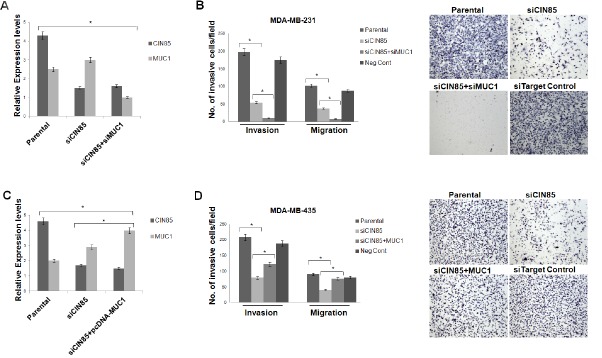
CIN85 and MUC1 co-regulate migratory and invasive properties of breast cancer cells (A and C) MDA-MB-231 cells were transfected with CIN85 and/or MUC1 siRNA whereas MDA-MB-435 cells were transfected with CIN85 siRNA and/or pcDNA-MUC1 plasmid. The graphs represent the CIN85 and MUC1 mRNA levels relative to GAPDH mRNA in the same sample as determined by Real-Time PCR. B) Cell invasion and migration were evaluated using a 24-well transwell migration assay and matrigel invasion chambers as illustrated in Material and Methods. The graphs show the ratio of the total number of invasive/migratory cells counted after 24h seeding in MDA-MB-231 transfected with siCIN85 and/or siMUC1 to the total number of cells not trasfected. D) The same assay was performed using MDA-MB-435 cells transiently transfected with siRNA and/or pcDNA-MUC1 plasmid. In control experiments the cells were transfected with non-targeting RNA siRNA (Neg Cont).

Co-localization on invadopodia and the apparent association with other molecules involved in cell migration and invasion suggested that MUC1 and CIN85 might play a role in those processes as well. We used siRNA targeting of CIN85 and achieved over 70% reduction of CIN85 mRNA in transfected MDA-MB-231 cells and another breast cancer cell line MDA-MB-435, compared to parental cells (Fig.6A and C). In a transwell invasion assay, siRNA-mediated knockdown of CIN85 suppressed migration of MDA-MB-231 cells by ~50% (Fig. 6B) and of MDA-MB-435 cells by ~45% (Fig. 6D). In a standard matrigel invasion assay, the silencing of CIN85 resulted in 75% and 65% reduction of invasion across the matrigel of MDA-MB-231 and MDA-MB-435 respectively (Fig. 6B and D). Next we asked if and how changes in MUC1 expression might modulate the role of CIN85 in migration and invasion. We observed that in MDA-MB-231 cells transfected simultaneously with MUC1 and CIN85 siRNA, migration and invasion were significantly reduced compared to siCIN85 transfected cells or control (nontargeting siRNA) samples (Fig. 6B). On the other hand, ectopic expression of MUC1 partially overcame this inhibition of migration and invasion induced by siCIN85 (Fig. 6D).

### Inhibition of tumor metastasis in vivo following downregulation of CIN85 is reversed by upregulation of MUC1

**Figure 7:**
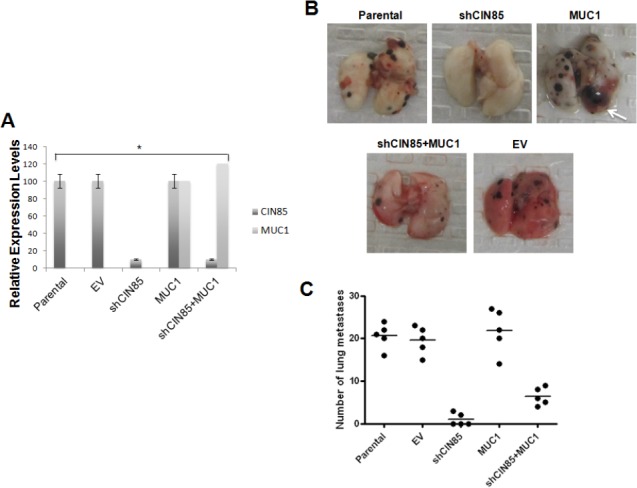
In vivo metastasis of B16 melanoma cells B16 (Parental) cells were transfected with shRNA for CIN85 (shCIN85), pcDNA-MUC1 plasmid (MUC1) or empty vector (EV). A) CIN85 and MUC1 protein levels in various B16 transfectants relative to GAPDH protein in the same sample as determined by Image J; B) Metastatic colonies in the lungs 14 days after intravenous injection of 8.5 × 10^4^ of various B16 transfectants; C) The number of colonies in the lung (n=5/group).

The data illustrated above, in agreement with other studies [17], showed that CIN85 is involved in the invasive activity of cancer cells. Next, we examined the role of CIN85 on the metastatic process and if this activity is regulated by MUC1. We used a well-characterized model of experimental lung metastasis produced by intravenous injection of B16 mouse melanoma cells. Parental B16 cells were stably transfected with shRNA CIN85 and MUC1 cDNA plasmids, and the successful transfections were confirmed by Western blotting (Fig. 7A). Next, the cells were injected (i.v.) into mice. The knockdown of CIN85 in shCIN85-transfected B16 cells (shCIN85) very effectively reduced the number of lung metastases compared with mice injected with untransfected parental cells: few or no metastatic lung nodules were detected (Fig. 7B and C).

MUC1 has been reported to enhance liver and lung metastasis in a mouse model of pancreatic cancer [5] and high MUC1 expression has been correlated with metastatic tumor phenotype in human cancer [23,24]. Our data showed that MUC1-transfected B16 cells (B16+MUC1) did not produce a significant difference in the number of lung metastases compared to untrasfected B16 cells. However, overexpression of MUC1 produced tumor of larger size compared to untrasfected B16 cells or the empty vector (EV) transfected cells, suggesting that MUC1 may modulate the rate of tumor growth (Fig. 7B, white arrow). Double transfection of shRNA CIN85 and MUC1 cDNA plasmids in B16 cells (B16+ shCIN85+MUC1) resulted in a significant reactivation of metastasis when compared to B16 cells transfected with shCIN85 alone. On the other hand, the volume of the tumors was significantly smaller than B16+MUC1-derived metastases.

Therefore, similarly to the *in vitro* data, these observations clearly indicate that CIN85 plays important roles in the promotion of tumor metastasis and growth and its activity is modulated by MUC1.

## DISCUSSION

MUC1 has been proposed to be a key modulator of several signaling pathways in epithelial cancers that affect tumor cell invasion, proliferation and survival [25-27]. This has supported its importance both as a diagnostic marker of poor prognosis and as a target for therapeutic intervention in cancer. The contribution of MUC1 to the invasive and metastatic properties of adenocarcinomas has been linked with some forms of MUC1 increasing the adhesive properties of tumor cells, and others promoting anti-adhesiveness (e.g. interacting with ICAM-1 and galectin-3), as well as by regulating cell signaling. MUC1 associates with β-catenin, NF-κB and EGFR and translocates to the nucleus thereby regulating transcription of several genes responsible for progression and invasiveness of cancer [6,28,29]. Here we report for the first time that CIN85 is a binding partner of MUC1 and implicate the MUC1/CIN85 complex in several of these functions. CIN85 is an adaptor protein involved in cellular processes such as ubiquitination, endocytic internalization and intracellular trafficking. CIN85 was reported to be highly expressed in human squamous cell carcinoma of the head and neck and in cervical carcinoma and this overexpression was significantly correlated with advanced clinical stages of disease [30,31].

Our results revealed that both the cytoplasmic tail and the extracellular domain of MUC1 interact (co-precipitate) with CIN85 and that the two molecules co-localize in the plasma membrane and cytosol, and associate with cytoskeletal proteins. Although further analyses are required, it is possible that CIN85 is responsible for MUC1 expression at the apical plasma membrane in normal cells and early stages of tumors and for its expression on the entire surface and in the cytosol in advanced stages of tumors. Significantly, we found that MUC1 and CIN85 co-localize at invadopodia-like protrusions. As the invasive capacity of cancer cells depends in part on their ability to assemble invadopodia [32–35], the formation of the MUC1/CIN85 complex may be a requirement for this process. We show that MUC1 and CIN85 co-localize with two known invadopodial proteins, cortactin and MT1-MMP. Cortactin was shown to regulate the formation of lamellopodia and invadopodia and MT1-MMP is a key enzyme that increases pericellular degradation of the matrix. The extent of their activity may be regulated by the relative presence or relative absence of the MUC1/CIN85 complex.

In this study, we show for the first time that B16 melanoma cells depend on both CIN85 and MUC1 to metastasize to the lung. In agreement with our *in vitro* data, our results showed that CIN85 activity is suppressed by MUC1 overexpression, indicating that CIN85 activity is modulated by MUC1. The presence or absence of the MUC1/CIN85 complex is dependent on the glycosylation state of the VNTR region in the extracellular domain of MUC1, which in turn is dependent on the transformation state of the cell. Aberrant glycosylation of MUC1 and overexpression of Tn- and sTn-MUC1 forms starts early in cancer development and gets progressively more abundant with fewer sugars added to the glycosylation sites as tumors progress to later stages. We propose that this leads to an increased interaction between MUC1 and CIN85 and thus an increase in the number of MUC1/CIN85 complexes that can promote the invasive activity of cancer cells. This is consistent with previous studies showing that the tandem repeats of MUC1, and in particular the alterations in the length of the glycan chains, play a role in cancer progression, invasiveness and attachment of carcinoma cells to tissues at distant sites [36-39]. This study provides new opportunities to use small molecule inhibitors or immunotherapy to target MUC1 and CIN85 association and thus potentially reduce cancer invasion and metastasis.

## MATERIALS AND METHODS

### Cell Culture

RMA mouse T cell lymphoma, IG-10 mouse melanoma cell line, B16 mouse melanoma cells and MDCK (Madin Darby Canine Kidney) cells were grown in DMEM (Dulbecco's Modified Eagle Medium, Cellgro, Mediatech, Inc., Herndon, VA, USA), and MDA-MB-231 and MDA-MB-435 human breast cancer cell lines and DM6 human melanoma cells were grown in RPMI-1640 (Royal Park Memorial Institute Medium, Cellgro) medium. Both DMEM and RPMI were supplemented with 10% heat-inactivated fetal bovine serum, 100 units/mL penicillin, 100 μg/mL streptomycin, 2 mmol/L L-glutamine. In vitro invadopodia formation was induced by treatment with 5 nM hEGF (Cell Signaling Technology, Beverly, MA, USA).

### Plasmids and Transfection

We utilized the pcDNA3 core plasmid (Invitrogen Life Technology, Carlsbad, CA, USA) into which we inserted (i) MUC1 cDNA containing 22 tandem repeats (MUC1/22TR), (ii) the short isoform MUC1/Y that lacks tandem repeats, (iii) chimeras of Tac (interleukin 2 receptor α subunit) and MUC1 including Tac-CT (cytoplasmic tail) and 22TR-Tac or the wild-type Tac that were previously described [18,19]. The cDNA for CMP-Neu5Ac:GalNAc-Rα 2,6- sialyltransferase-1 (ST6GalNAc-1) and ST3 β-galactoside α-2,3-sialyltransferase 1 (ST3Gal1) were also subcloned into pcDNA3 [19]. CIN85 shRNA plasmid was purchased from Santa Cruz. C2GalT1 HuSH-29 pre-designed shRNA for human C2GalT1 enzyme was purchased from Origene (OriGene Technologies, Inc, Rockville, MD, USA). Transfections were performed with Lipofectamine 2000 (Invitrogene Life Technology) according to manufacturer's instruction.

MUC1, CIN85 or control siRNAs were purchased from Dharmacon (SMARTpools, Dharmacon, Lafayette, CO). The siRNA transfections were performed with X-tremeGENE siRNA transfections (Roche Applied Science, Indianapolis, IN, USA) as per the manufacturer's specifications. Transient transfections were achieved for 48-96h whereas stable expression was achieved by selection in up to 1000 μg/mL G418 (Sigma-Aldirich), 2000 μg/mL puromycin (Santa Cruz) or 1000 μg/mL Hygromycin (Invitrogene Life Technology).

### Western blotting and Immunoprecipitation

Total cell proteins were extracted using RIPA buffer (150 mM NaCl, 0.5 sodium deoxycholate, 0.1 % SDS, 1% NP-40 and 50 mM Tris-HCl,) with commercial protease inhibitors (Complete Protease Inhibitor Cocktail from Roche, Mannheim, Germany) and phosphatase inhibitors (Phosphatase Inhibitor Cocktail II, Sigma-Aldirich).

Subcellular fractions were prepared using Subcellular Protein Fractionation Kit (Thermo Scientific, Rockford, IL, USA) following the manufacturer's instructions.

50 μg of protein from lysates was subjected to SDS-PAGE for Western blotting (WB), while 500 μg was used for immunoprecipitation (IP). The following antibodies were employed: anti-CIN85 (A-7), anti-CIN85 (H300), anti-actin (Santa Cruz Biotechnology, Santa Cruz, CA, USA) for WB and IP; anti-MUC1 (VU-3C6, a gift from Dr. Hilgers, Free University, Brussels), anti-MUC1 4H5 (Santa Cruz) or anti-MUC1 (Ab5) (Fitzgerarld, Concord, MA, USA) for WB and IP, anti-Tac (Ancell, Bayport, MN, USA).

For immunoprecipitation, protein lysates were first pre-cleared with Protein G-Sepharose beads (Sigma-Aldrich, St Louis, MO, USA) for 3h at 4 °C, then incubated with primary antibody at 4 °C for 16 h. The immune complexes were precipitated for 2 h at 4 °C with Protein-G Sepharose 4B. In control samples, the primary Ab was substituted with non-immune IgG (rabbit or mouse, depending on the source of the primary Abs). Immunoprecipitates were washed six times with RIPA lyses buffer containing 0.5 M NaCl, 2% sodium dodecyl sulfate (SDS), 2 times with PBS, then were resuspended in Laemmli Buffer.

The proteins were separated on a pre-cast 4-20 % polyacrylamide gel (BioRad, Hercules, CA, USA) immunoblotted, and immunoblots were developed with horseradish peroxidase-conjugated secondary antibody and chemoluminescence reagents (Perkin-Elmer Life Sciences, Boston, MA, USA). Intensity of signals was determined by densitometric scanning (Kodak, Image Station 4000MM).

### Proteomics analysis

MUC1 immunoprecipitates were separated on a pre-cast 4-20 % polyacrylamide gel (BioRad), stained with Comassie Blue and washed with distilled water. Bands were cut from gels and eluted proteins analyzed by Maldi-Tof (Spectometry Mass, Genomic and Proteomic, University of Pittsburgh).

### Quantitative real-time PCR

Total RNA was isolated using RNeasy Mini Kit (Qiagen, Valencia, CA, USA) according to manufacturer's instructions. A total of 2 μg of RNA was reverse transcribed using the SuperScript First Strand Kit (Invitrogen). A total of 3 μl of RT products were used to amplify CIN85, MUC1 and GAPDH as an internal control. Real-time PCR was performed using the SYBR Green PCR kit (Qiagen) and the Applied Biosystem StepOnePlus Real-Time PCR (Applied Biosystem, Foster City, CA, USA). Changes in the mRNA content relative to GAPDH mRNA were determined using a threshold cycle (CT) method (ABI User Bulletin no. 2) to calculate changes in CT and, ultimately, fold and percent change. An average CT value for each RNA was obtained for replicate reactions.

### Immunohistochemistry

Human breast cancer tissue paraffin sections (Biochain, Newark, CA, USA) were deparaffinized by baking overnight at 59°C. Endogenous peroxidase activity was eliminated by treatment with 30% H_2_O_2_ for 15 min at room temperature. Antigen retrieval was performed by microwave heating in 0.1% citrate buffer. Nonspecific binding sites were blocked with Protein Blocking Agent (Thermo-Shandon). The anti-MUC1 antibody 4H5, which recognizes the epitope APDTRPAP in the VNTR region of hypoglycosylated MUC1, was purchased from Santa Cruz Biotechnology. The anti-CIN85 (anti-SH3KBP1) antibody was purchased from Novus Biologicals (Littleton, CO, USA). Staining was performed by the avidin-biotin-peroxidase complex method with a commercial kit (Vectastain ABC kit; Vector Laboratories, Burlingame, CA, USA). Positive signals were visualized by a DAB Kit (BD Pharmingen, San Jose, CA, USA).

### Immunofluorescence confocal microscopy

Cells were fixed with 4% paraformaldehyde for 20 min and permeabilized in 0.5% Triton-X100 for 20 min. The fixed cells were incubated with anti-MUC1 4H5, anti-CIN85 (Santa Cruz), anti-cortactin (EMD Millipore Corporation, Billerica, MA, USA), anti-MT1-MMP (Calbiochem), FITC-labeled mouse anti-human MUC1 antibody CD227 (BD Pharmingen), for 2 h at RT followed by Cy3 secondary anti-mouse Alexa-467 or Alexa-488 antibody (Invitrogen Life Technology). Nuclei were stained with mounting medium with DAPI (VectorLab).

### Migration and invasion assay

*In vitro* migration studies were conducted using a transwell (Boyden Chamber) assay (Costar Transwell Assay; Corning Inc., Corning NY). Cells (10^4^ cells per well) were plated in the upper chamber in Serum Free Medium. 600 μl of 10% FBS containing medium was placed in the lower chamber. After 16 h cells were fixed with 4% paraformaldehyde for 20 min and stained with Christal Violet 1% (Siemens Inc., Deerfield, IL). Cells adhering to the top of the filter were gently wiped away and those adhering to the lower surface of the filter were counted using a microscope at 100X magnification (10 fields). For invasion assays, cells were allowed to migrate through a filter pre-coated with matrigel (BD Biocat, BD Biosciences, Two Oak Park, Bedford, MA), fixed and counted as above.

### In vivo Metastasis Assay

Six- to 8-week old C57BL/6 mice were purchased from The Jackson Laboratory (Bar Harbor, ME). 8.5 X10^4^ B16, B16/shCIN85, B16/MUC1 or B16/shCIN85+MUC1 or B16 cells transfected with a vector control were injected (i.v.) into mice *via* the lateral tail vein. Two weeks later, the mice were sacrificed, the lungs were fixed in 10% neutral buffered formalin, and the black melanoma nodules on the lungs were quantified. All mice were maintained at the University of Pittsburgh Animal Care Facility under specific pathogen free conditions. Experiments were approved by the Institutional Animal Care and Use Committee of the University of Pittsburgh.

### Statistical analysis

The correlations were evaluated by two-tailed Student's *t* test. *P* values of <0.05 were considered statistically significant.

### Supplementary Figures


