# Supplementary material for: Altered glycosylation of MUC1 influences its association with CIN85: the role of this novel complex in cancer cell invasion and migration
Source: Oncotarget. 2013 Sep 7;4(10):1686–97. doi: 10.18632/oncotarget.1265 (PMC3858555; doi:10.18632/oncotarget.1265)
Supplement: Supplementary file 1 [file oncotarget-04-1686-s001.pdf]

Altered glycosylation of MUC1 influences its association with CIN85: the role of this novel complex in cancer cell invasion and migration - Cascio et al

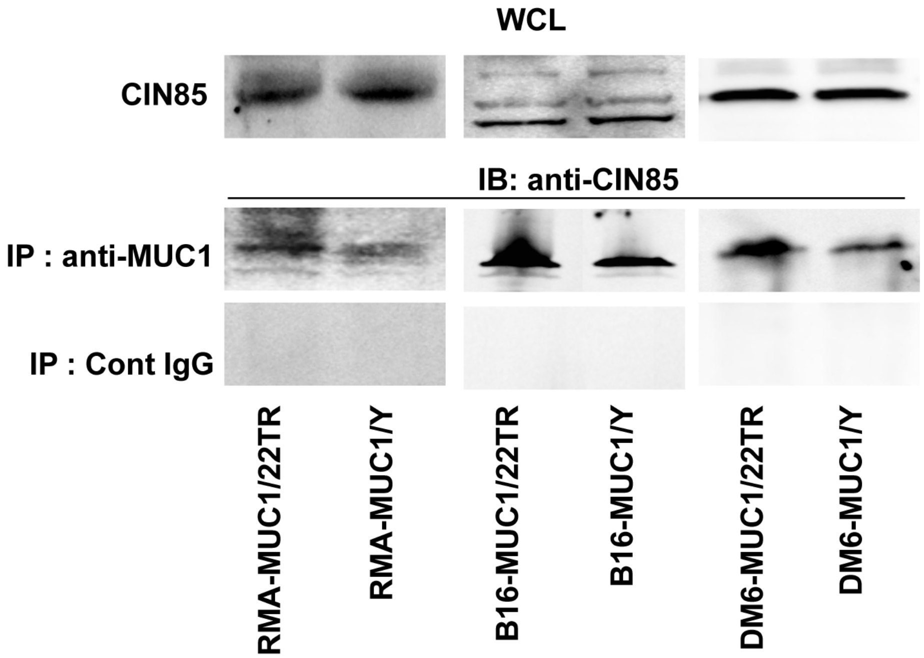

Supplemental Data 1: Whole cell lysate (WCL) from MUC1/22TR and MUC1/Y stably transfected in RMA, B16 and DM6 cells was immunoblotted with anti-CIN85 (upper panel) or immunoprecipitated with anti-MUC1 Ab5 or a control IgG and immunoblotted with anti-CIN85 (lower panel).

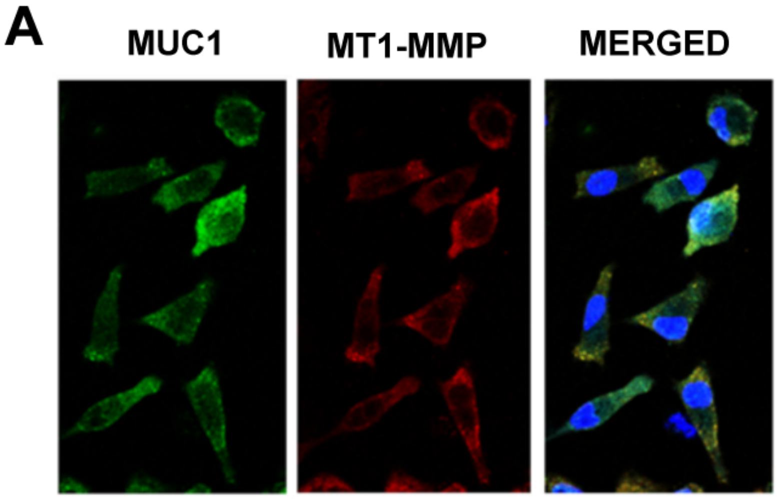

Supplemental Data 2: Confocal immunofluorescence microscopy. MDA-MB-231 breast cancer cells were fixed and stained with anti-MUC1 (4H5) (green), anti- MT1-MMP (red). Nuclei were stained with DAPI (blue)
